# Radiographic prediction model based on X-rays predicting anterior cruciate ligament function in patients with knee osteoarthritis

**DOI:** 10.1186/s42492-025-00195-w

**Published:** 2025-06-06

**Authors:** Guanghan Gao, Yaonan Zhang, Lei Shi, Lin Wang, Fei Wang, Qingyun Xue

**Affiliations:** 1 Department of Orthopedics, Beijing Hospital, National Center of Gerontology, Institute of Geriatric Medicine, Chinese Academy of Medical Sciences & Peking Union Medical College, Beijing, 100730 China; 2 Department of Orthopedics, Beijing Hospital, National Center of Gerontology, Institute of Geriatric Medicine, Chinese Academy of Medical Sciences, Beijing, 100730 China

**Keywords:** Knee osteoarthritis, X-ray, Anterior cruciate ligament, Prediction model

## Abstract

Knee osteoarthritis (KOA) is a prevalent chronic condition in the elderly and is often associated with instability caused by anterior cruciate ligament (ACL) degeneration. The functional integrity of ACL is crucial for the diagnosis and treatment of KOA. Radiographic imaging is a practical diagnostic tool for predicting the functional status of the ACL. However, the precision of the current evaluation methodologies remains suboptimal. Consequently, we aimed to identify additional radiographic features from X-ray images that could predict the ACL function in a larger cohort of patients with KOA. A retrospective analysis was conducted on 272 patients whose ACL function was verified intraoperatively between October 2021 and October 2024. The patients were categorized into ACL-functional and ACL-dysfunctional groups. Using least absolute shrinkage and selection operator regression and logistic regression, four significant radiographic predictors were identified: location of the deepest wear on the medial tibial plateau (middle and posterior), wear depth in the posterior third of the medial tibial plateau (> 1.40 mm), posterior tibial slope (PTS > 7.90°), and static anterior tibial translation (> 4.49 mm). A clinical prediction model was developed and visualized using a nomogram with calibration curves and receiver operating characteristic analysis to confirm the model performance. The prediction model demonstrated great discriminative ability, showing area under the curve values of 0.831 (88.4% sensitivity, 63.8% specificity) and 0.907 (86.1% sensitivity, 82.2% specificity) in the training and validation cohorts, respectively. Consequently, the authors established an efficient approach for accurate evaluation of ACL function in KOA patients.

**Supplementary Information:**

The online version contains supplementary material available at 10.1186/s42492-025-00195-w.

## Introduction

Knee osteoarthritis (KOA), a common chronic disease in the elderly, often exhibits highly consistent patterns of onset, progression, and prognosis [1, 2]. The degeneration of anterior cruciate ligament (ACL) is the major cause of KOA [3]. ACL is a key ligament guiding knee joint movement and resisting anterior tibial translation and rotational loads. It is one of the most frequently injured structures during high-impact or athletic activities [4–6]. Unfortunately, ACL tears often fail to heal [7], and its injury, degeneration, and rupture are significant contributors to the worsening of KOA [8, 9]. Accurate assessment of ACL function in patients with KOA is crucial for evaluating disease progression and determining appropriate indications for knee arthroplasty such as Oxford unicompartmental knee arthroplasty (UKA) [10] and bicruciate-retaining total knee arthroplasty (TKA) [11]. Therefore, there is an urgent need for effective methods to evaluate ACL function in KOA patients.

Currently, magnetic resonance imaging (MRI) remains the standard for directly visualizing ACL injuries; however, its ability to evaluate ACL function is sometimes limited owing to over-sensitive imaging findings [8, 9, 12–14]. ACL dysfunction changes the movement pattern of a normal knee joint and forms some special imaging signs of KOA. Notably, when ACL dysfunction occurs, wear may extend to the posterior tibial plateau or manifest as posterior subluxation of the femur [9, 10]. Owing to osseous structural abnormalities, X-ray diagnostics offer a distinct advantage in identifying these radiographic signs. In addition, radiographs avoid misleading false-positive results for ACL injury assessment using MRI [12]. Therefore, the Oxford UKA manual recommends evaluating ACL function in KOA patients using lateral radiographic imaging rather than MRI [12, 15, 16].

Radiographic imaging has been used to predict the functional status of the ACL [12, 16–18]. However, the evaluation accuracy of these studies is generally limited by small sample size, lack of effective validation, and insufficient inclusion of imaging features of interest. Intraoperative assessment of ACL function is the most direct diagnostic method compared with other imaging techniques. Therefore, we must develop a new predictive method to avoid these drawbacks and enhance the accuracy of radiographic diagnosis for ACL function.

In this study, we extracted four radiographic features from a large patient cohort and constructed a prediction model and scoring system for ACL function using a nomogram. The ACL function verified through direct intraoperative assessment. Our prediction model provides an accurate method for predicting the ACL function of KOA patients using preoperative radiographs, thereby guiding physicians in making decisions regarding surgical indications or follow-up treatment.

## Methods

### Study design and participants

This retrospective study included patients who underwent primary knee arthroplasty at our center between October 2021 and October 2024. A total of 272 KOA patients who had complete preoperative radiographs (anteroposterior, lateral, and full-length lower limbs) were enrolled, and their ACL function assessed and recorded during surgery were included. Patients diagnosed with rheumatoid arthritis, neuromuscular diseases, a history of knee trauma, or insufficient preoperative radiographs were excluded. All patients were randomly divided into training and validation cohorts at a ratio of 7:3 using stratified sampling. All the surgeries were performed by the same team of surgeons. This study was approved by the Beijing Hospital Ethics Committee (2018BJYYEC-031-01), and informed consent was obtained from all enrolled patients.

### ACL function assessment

The surgeon evaluated the ACL function intraoperatively during knee arthroplasty. After adequate exposure to the surgical field, the ACL was inspected. In cases where the ACL was absent, the patient was assigned to the ACL-dysfunctional (ACLD) group. In cases where the ACL was present, a small retractor or vascular clamp was used to measure the tension. In cases where the ACL lost tension and became lax, deformed, or ruptured under traction, the patient was also assigned to the ACLD group. In cases in which the ACL exhibited elasticity, and tension was restored after traction, the patient was assigned to the ACL-functional (ACLF) group (Supplementary Fig. S1).

### Radiographic features extraction

All radiographic films were prepared in the Radiology Department of our hospital using the same equipment (Siemens Healthineers, Germany). To obtain standardized radiographs for measurement purposes, we selected the radiographs to be included in the study based on the following criteria. For anteroposterior views, the tibial plateau was aligned to obscure one-third of the fibular head without any anterior or posterior slope. The lateral views were obtained by overlapping the medial and lateral condyles of the femur. These standardized radiographs mitigated the impact of knee rotation on measurement accuracy. The distances required for the measurements were calculated using a reference scale on the radiographs. Based on previous studies and our clinical experience, we selected the following radiographic variables that may influence the assessment of ACL function.

For anteroposterior radiographs, the following features were measured or observed.

1. Kellgren-Lawrence (K-L) classification for KOA (Grade III or IV). Grade III is characterized by multiple osteophytes, definite narrowing of the joint space, some sclerosis, and possible deformity of the articular surface. Grade IV is defined by large osteophytes, marked narrowing of the joint space, severe sclerosis, and definite deformity of articular surface.
2. Intercondylar osteophyte (IO): with or without IO.
3. Coronal tibiofemoral subluxation (CTFS): the distance between the tangent line to the lateral edge of the femoral condyle and the tangent line of the lateral tibial plateau [19, 20].
4. Tibial spine sign (TSS): three types of standardized anteroposterior standing knee radiographs–type 1: no contact between the lateral femoral condyle and the lateral tibial intercondylar spine; type 2: contact between the lateral femoral condyle and lateral tibial intercondylar spine; type 3: overlap between the lateral femoral condyle and lateral tibial intercondylar spine [21].

For lateral radiographs:

1. Location of the deepest wear on the medial tibial plateau (position): anterior, middle, or posterior (the tibial plateau was divided into three equal parts).
2. Maximum depth of wear on the medial tibial plateau (D/mm): the distance from the tibial plateau tangent to the deepest point of wear.
3. Depth of wear in the anterior one-third of the medial tibial plateau (D1/mm): the distance from the tibial plateau tangent to the wear point at the anterior third of the tibial plateau.
4. Depth of wear in the posterior third of the medial tibial plateau (D2/mm): the distance from the tibial plateau tangent to the wear point at the posterior third of the tibial plateau.
5. Medial tibial osteophyte (TO): with or without medial TO.
6. Posterior femoral osteophyte (FO): with or without posterior FO.

For full-length lower limb radiographs:

1. Hip-knee-ankle angle (HKA): the angle between the mechanical axis of the tibia and the femur.
2. Posterior tibial slope (PTS): the angle between a line perpendicular to the tibial diaphysis and the medial tibial plateau [22].
3. Static anterior tibial translation (SATT): the distance between two lines parallel to the posterior tibial cortex; the first line is tangent to the posterior aspect of the medial tibial plateau, and the second line is tangent to the posterior femoral condyles [23, 24].

**Fig. 1:**
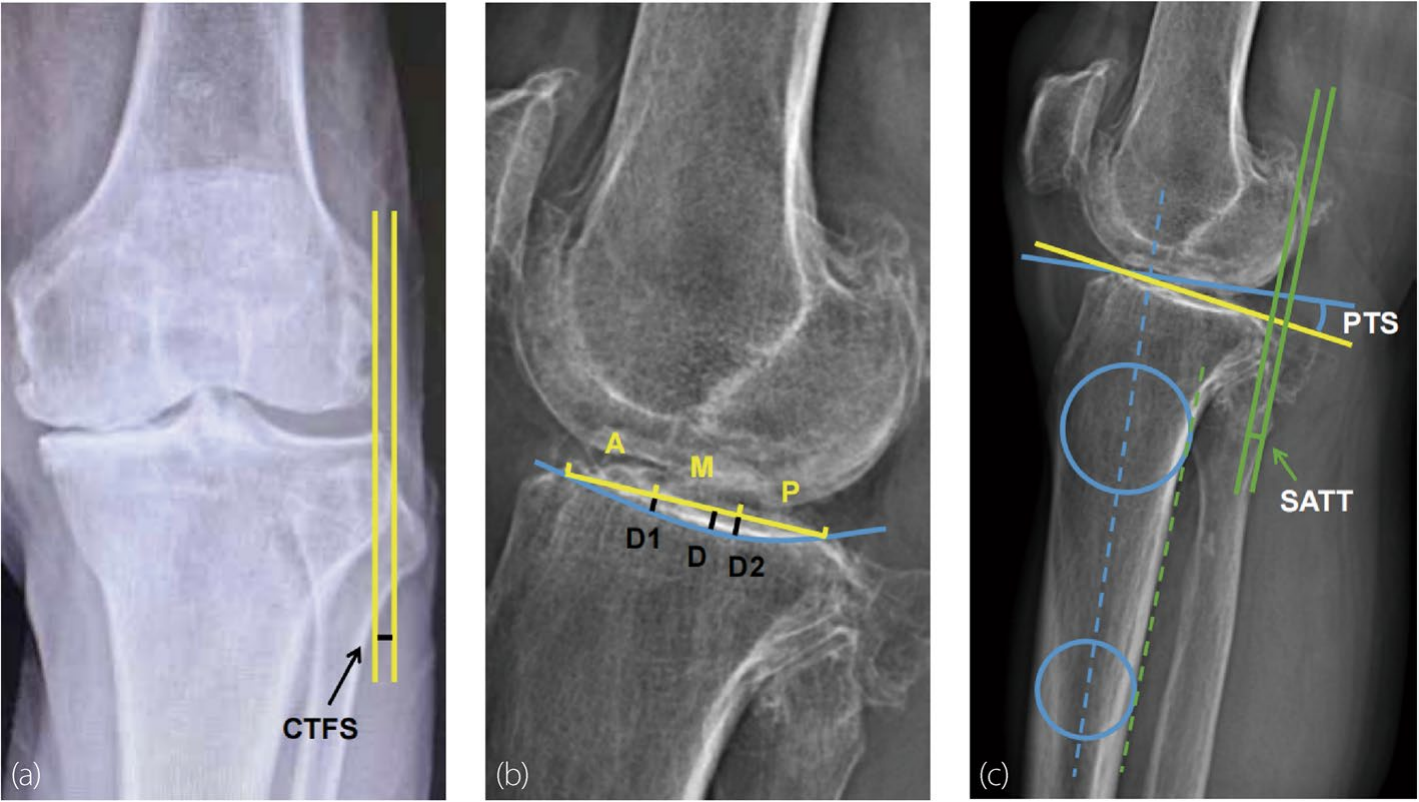
Assessment of radiographic features. **a** Anteroposterior X-ray. CTFS was defined as the distance between the tangent line (yellow solid line) to the outermost joint edge of the lateral condyle of the femur and the tangent line of the lateral tibial plateau; **b** Lateral X-ray. Medial tibial plateau was divided into three parts: anterior (A), middle (M) and posterior (P). D1 and D2 were measured at the three equidistant points along the medial tibial plateau. D was the maximum depth of wear on the medial tibial plateau; **c** Full-length lower limb X-ray. PTS was the angle formed between a line (blue solid line) perpendicular to the tibial diaphyseal axis (blue dotted line) and a line (yellow solid line) tangent to the most superior points at the anterior and posterior edges of the medial plateau. SATT was the distance between two lines (green solid lines) which were tangent to the posterior part of the medial plateau and medial femoral condyle, paralleling to the posterior tibial cortex (green dotted line)

All measurements were individually performed by two orthopedic surgeons using the hospital’s Picture Archiving and Communication System (PACS, Neusoft, China) (Fig. 1). The intra-class correlation coefficient (ICC) was used to assess inter-observer reliability for continuous variables (CTFS, D, D1, D2, HKA, PTS, and SATT), whereas Cohen’s kappa coefficient (κ) was used for nominal variables (K-L, TSS, position, IO, TO, and FO). For continuous variables, the results were determined by averaging the measurements from both observers. For categorical variables, in cases of disagreement between the two researchers, a senior orthopedic surgeon was consulted to assist in the diagnosis, and a consensus among the three was adopted. All researchers underwent standardized measurement training prior to the study and were blinded to the intraoperative assessment of ACL functional outcomes.

### Development of clinical model, radiomics signature and nomogram

Least absolute shrinkage and selection operator (LASSO) regression compresses the coefficients of insignificant features to zero, thereby enabling automatic variable selection, which makes it highly useful for feature selection. Therefore, we used it to model and screen radiographic variables for their importance. The dependent variable was the ACL function and the independent variables were position, D, D1, D2, TO, FO, IO, K-L, CTFS, PTS, HKA, SATT, and TSS. LASSO regression revealed that the model was most simplified and efficient when lambda was chosen to have one standard error. Features with non-zero LASSO coefficients were retained. Further selection was performed by using a logistic regression model. Odds ratios (ORs) and confidence intervals (CIs) were calculated to assess the associations between variables and ACL function. A nomogram is a graphical tool built based on a multifactorial regression analysis. It integrates multiple predictive indicators and represents them using scaled line segments drawn proportionally on a single plane, thereby illustrating the interrelationships among the variables in a predictive model. A radiographic nomogram was used to visualize the predicted model according to the selected features and generate numerical probabilities for ACLD. A multicollinearity analysis was performed for all independent radiographic variables.

 Receiver operating characteristic (ROC) and area under the curve (AUC) were used to determine the diagnostic accuracy of the logistic regression model. The optimal cutoff value was obtained using the maximum Youden index to balance sensitivity and specificity. When the radiographic variables surpass this cutoff value, it indicates that the patient has a higher likelihood of being diagnosed with ACLD. Calibration curves were constructed to depict the calibration of the nomogram in the training and validation cohorts.

### Statistical analysis

**Fig. 2:**
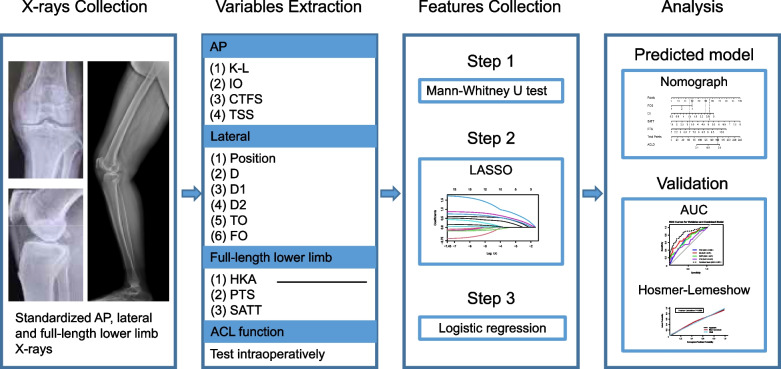
The workflow of this study. This study comprised four parts: X-ray data collection, imaging variable extraction, radiographic feature collection and screening, and predictive analyses

Continuous variables are presented as means ± SD, whereas categorical variables are presented as frequencies and percentages. The Shapiro-Wilk normality test was used to assess the distribution of continuous variables. The Mann-Whitney U test and chi-square test were performed for continuous and categorical variables, respectively. All statistical analyses were performed using R (version 4.4). Statistical significance was considered at* P* < 0.05, with the following designations: *P < 0.05 and **P < 0.01. Figure 2 illustrates the workflow of this study.

## Results

### Patient enrolment

A total of 318 patients satisfied the inclusion criteria. Among them, six were excluded due to rheumatoid diseases, four had a history of knee trauma, and 36 did not satisfy the study requirements. Therefore, 272 patients were included in this study (a flowchart of patient selection is shown in Supplementary Fig. S2). The average age was 69.8 ± 6.6 years, and the average body mass index (BMI) was 26.3 ± 3.2 kg/m^2^. Of these, 90 patients underwent UKA and 182 patients underwent TKA. The ACLF and ACLD groups comprised of 150 and 122 patients, respectively (Table 1).

**Table 1:** Basic characteristic

Group		Age (year)	*P*	Gender, No. (%)		*P*	BMI (kg/m^2^)	*P*	Side, No. (%)		*P*
				Male	Female				Left	Right	
Training	ACLF (*n* = 105)	69.4 ± 6.4	0.110	51 (48.6%)	54 (51.4%)	0.055	27.1 ± 3.4	0.032	55 (52.4%)	50 (47.6%)	0.732
	ACLD (*n* = 86)	70.8 ± 6.2		29 (33.7%)	57 (66.3%)		26.0 ± 3.0		42 (48.8%)	44 (51.2%)	
Validation	ACLF (*n* = 45)	66.6 ± 5.8	< 0.01	23 (51.1%)	22 (48.9%)	0.672	25.8 ± 3.2	0.383	19 (48.8%)	26 (51.2%)	0.470
	ACLD (*n* = 36)	72.6 ± 7.4		21 (58.3%)	15 (41.7%)		25.2 ± 3.0		19 (52.8%)	17 (47.2%)	

### Nomogram variable screening

**Fig. 3:**
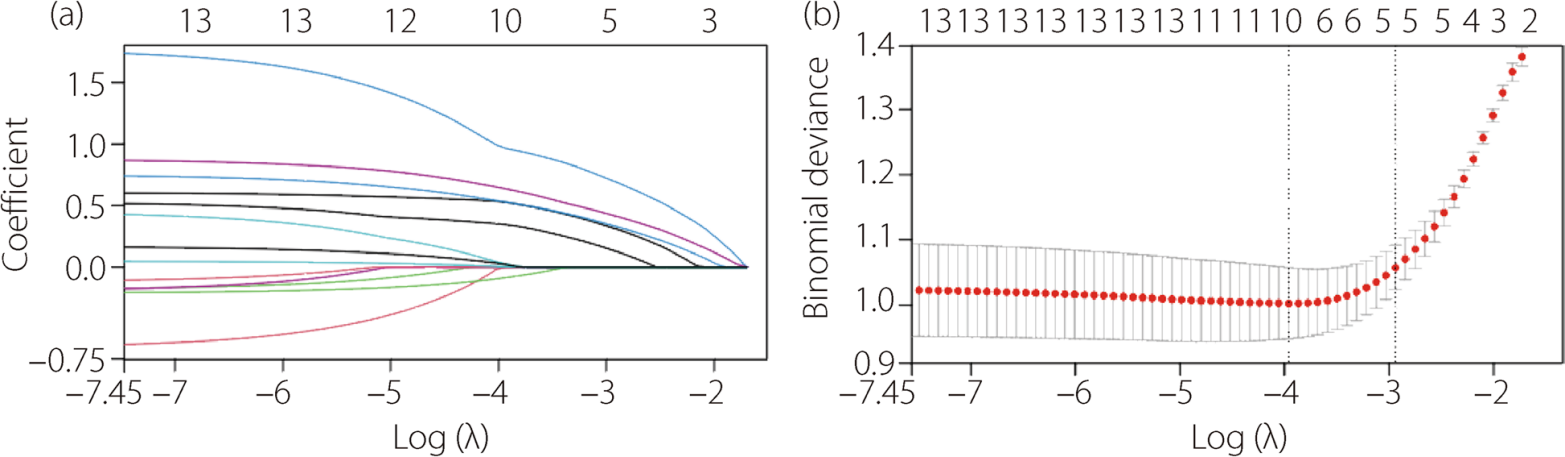
Radiographic variables were selected by LASSO model in the general population. **a** LASSO coefficients of radiographic features; **b** Tuning parameter (λ) selection in the LASSO model used tenfold cross-validation via minimum criteria

**Fig. 4:**
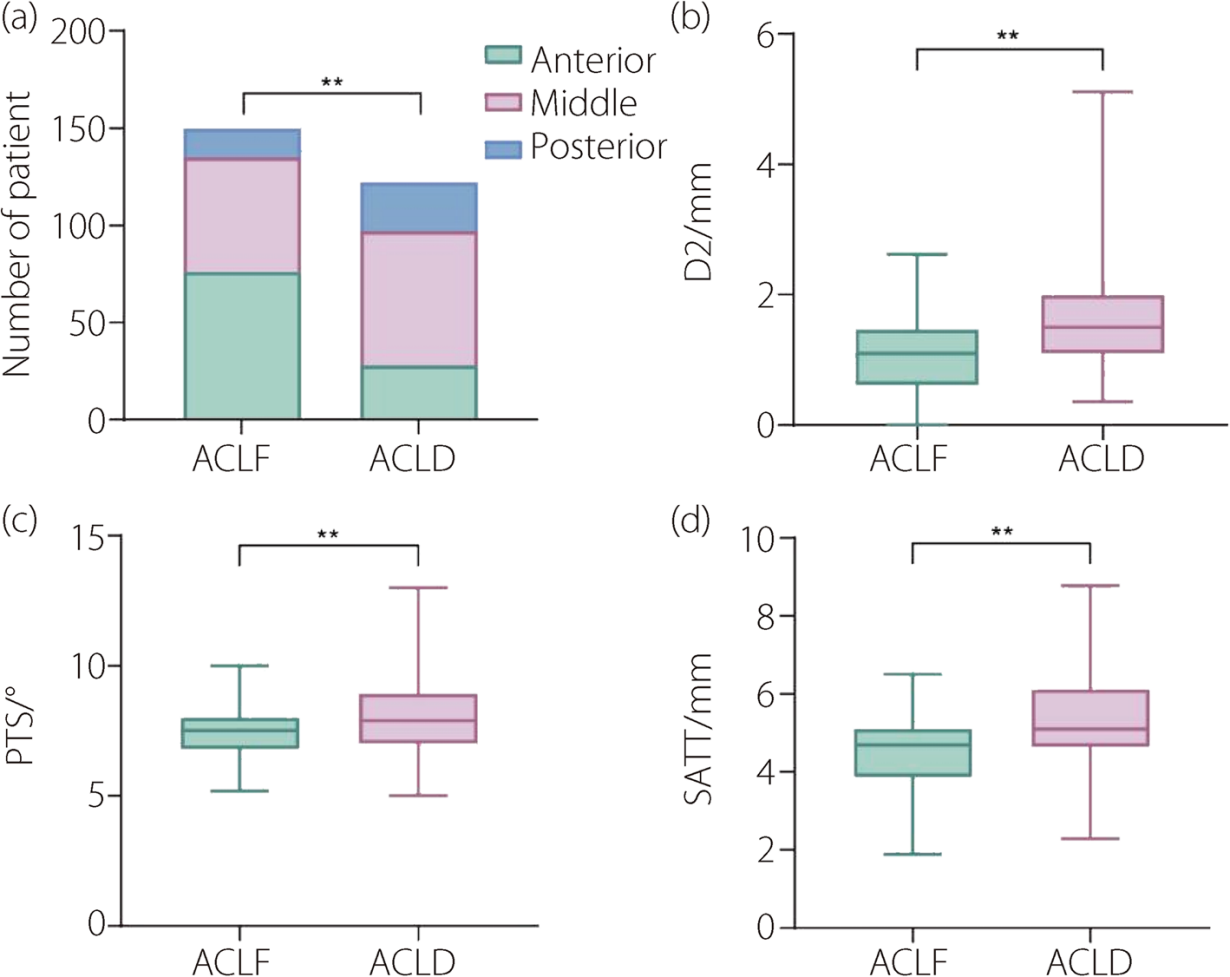
The differences in position (**a**), D2 (**b**), PTS (**c**) and SATT(**d**) between ACLF and ACLD group in the general population

We extracted 13 factors from the radiograph image data of all patients. LASSO regression was performed to screen for contributing factors. Position, D2, IO, PTS, and SATT were the most valuable predictors of ACL function (Fig. 3). However, in the logistic model, IO was no longer significantly associated with ACL dysfunction (Table 2). In addition, position, D2, PTS, and SATT also showed statistically significant differences between ACLF and ACLD groups (Fig. 4). Compared to the anterior group, the ORs for the middle and posterior groups were 2.442 and 3.825, respectively, both of which were statistically significant. The ORs for D2, PTS, and SATT were 3.397, 2.014, and 2.201, respectively, indicating statistical significance. The ICCs or kappa coefficients (κ) for the radiographic variables were all greater than 0.8, demonstrating good inter-observer reliability (Table 2). No multicollinearity was found among the radiographic variables (the variance inflation factor values for all variable were below 5). Thus, position, D2, PTS, and SATT were valuable predictors of ACL function and were selected for nomogram construction. Based on the principle of events per variable, each variable had at least ten outcome events [25]. Therefore, the sample size of this study was adequate. The logistic regression model representing the probability of ACLD is as follows:

$$p=\frac1{1+e^{-\left(-11.985+0.967\;POS\left(1\right)+1.420\;POS\left(2\right)+1.275\;D2+0.833\;SATT+0.688\;PTS\right)}}$$

**Table 2:** LASSO and logistics regression analysis of radiographic predictors

	LASSO coefficient	OR (95%CI)	*P*	ICC or κ
Position	0.309		< 0.01	0.935
Anterior		1		
Middle		2.442 (1.213, 4.918)	0.012	
Posterior		3.825 (1.482, 9.867)	< 0.01	
D2	0.690	3.397 (1.891, 6.102)	< 0.01	0.836
IO	0.127	1.654 (0.883, 3.099)	0.116	0.912
PTS	0.331	2.014 (1.535, 2.643)	< 0.01	0.876
SATT	0.414	2.201 (1.603, 3.021)	< 0.01	0.821

### Nomogram construction and validation

**Fig. 5:**
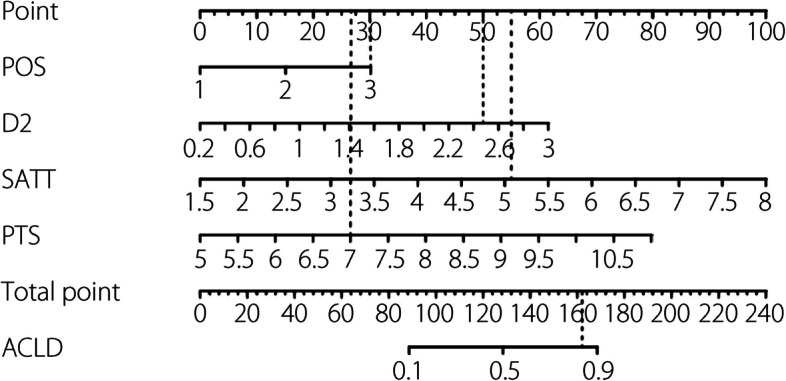
The combined prediction model visualized using a nomogram based on training cohort. One patient, with a total score of 162, was intraoperatively diagnosed with ACLD

Subsequently, we constructed a radiographic nomogram for ACL dysfunction based on the four predictors selected using LASSO and logistic regression. Each predictor with a given value was mapped onto a point axis. The sum of points was determined from the total point axis. The risk of ACL dysfunction was determined from the scale in the last row by vertically drawing a line from the total points. Taking a patient scheduled for knee replacement surgery as an example, measurements from the radiographic images revealed that the deepest wear was located at the posterior aspect of the tibial plateau, with D2 measuring 1.40 mm, SATT was 5.09 mm, and PTS was 7.0°. The total score calculated using the nomogram was 162 points, indicating that the patient’s risk of ACLD exceeded 0.8. Therefore, after communicating with the patient, we opted for TKA, which is suitable for ACLD cases, rather than UKA. Intraoperative examination confirmed the patient’s ACLD condition (Fig. 5).

**Fig. 6:**
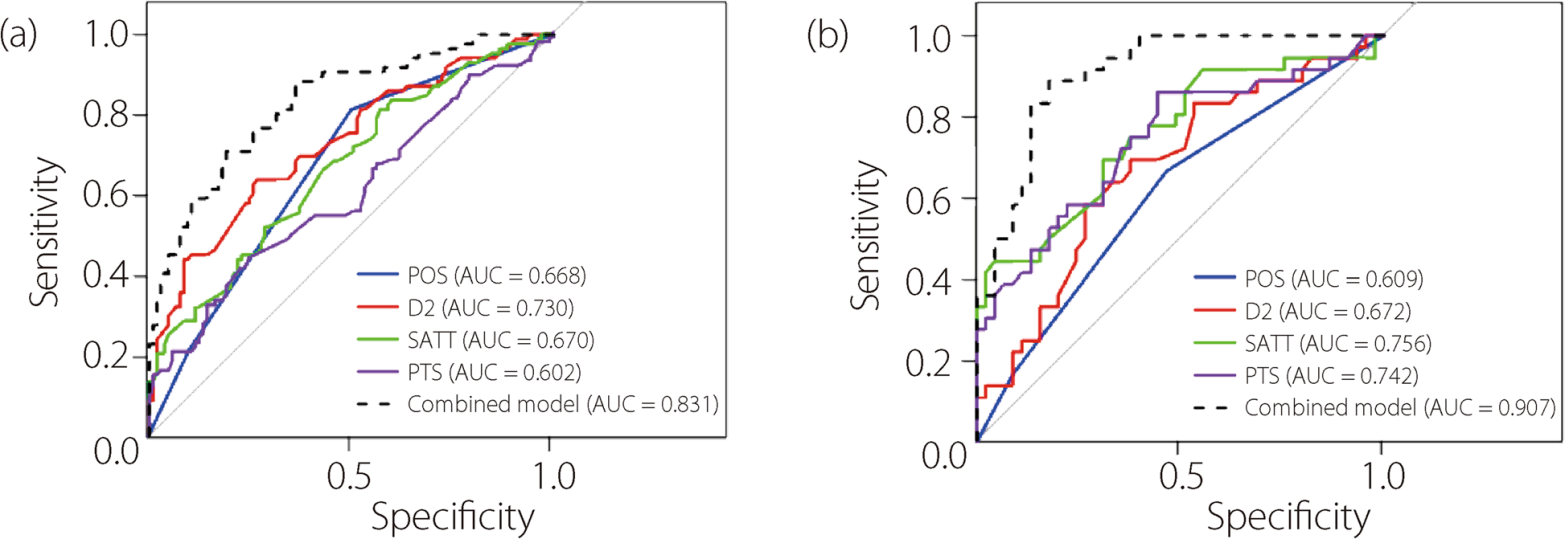
ROC curves for predictors and combined model in different cohorts. The AUC of the combined model were 0.831 in the training cohort (**a**) and 0.907 in the validation cohort (**b**)

Subsequently, ROC curves of the predictors and nomogram were used to confirm the diagnostic accuracy of the prediction model (Fig. 6). The AUCs of each predictor and nomogram were all higher than 0.6, which were statistical significant, except for the AUC of the position in the validation cohort (AUC = 0.609, *P* = 0.093). The AUCs of the nomogram were 0.831 (sensitivity, 88.4%; specificity, 63.8%) and 0.907 (sensitivity, 86.1%; specificity, 82.2%) for the training and validation cohorts, respectively. The cutoff values were anterior-middle for the position (A–M), 1.40 mm, 7.90°, and 4.49 mm in the training cohort, which were similar in the validation cohort (Table 3).

**Table 3:** AUCs and cutoff values of the prediction model for ACL dysfunction

Variable	Group	AUC	Cutoff	Sensitivity	Specificity	*P*
Position	Training	0.668	A–M^a^	81.4%	49.5%	< 0.01
	Validation	0.609	A–M^a^	66.7%	53.3%	0.093
D2	Training	0.730	1.40 mm	64.0%	73.3%	< 0.01
	Validation	0.672	1.37 mm	58.3%	73.3%	< 0.01
PTS	Training	0.602	7.90°	44.2%	75.2%	0.015
	Validation	0.742	7.87°	72.2%	64.4%	< 0.01
SATT	Training	0.670	4.49 mm	81.4%	42.9%	< 0.01
	Validation	0.756	4.87 mm	69.4%	68.9%	< 0.01
Model	Training	0.831	0.32	88.4%	63.8%	< 0.01
	Validation	0.907	0.36	86.1%	82.2%	< 0.01

^a^The middle of the anterior and middle groups in position. This indicates that patients in the middle and posterior groups were more likely to have ACL dysfunction

**Fig. 7:**
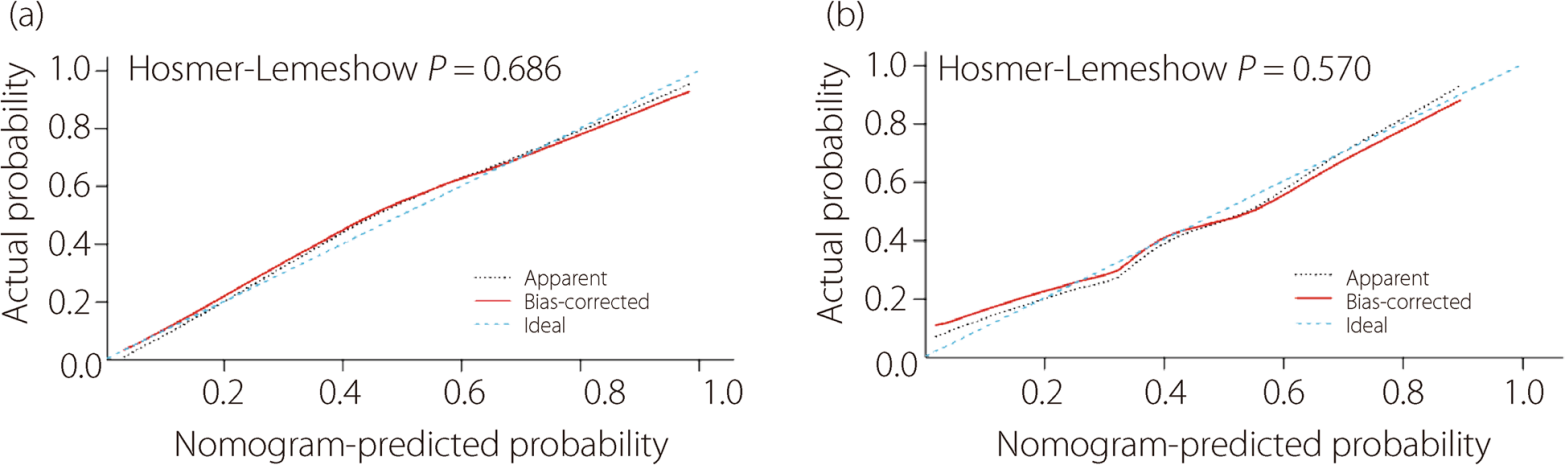
Calibration curves for nomogram in different cohorts. The calibration curves showed good predictive efficiency in the training cohort (**a**) and validation cohort (**b**), with *P* value of the Hosmer-Lemeshow test > 0.05

Calibration curves using the Hosmer-Lemeshow test for the training and validation cohorts indicated acceptable agreement between the nomogram-predicted probability and the actual probability of ACL dysfunction. The red solid line and black dotted line are close to the blue dotted line representing an ideal diagnosis, which indicates the accuracy of the nomogram (Fig. 7). In summary, the nomogram for ACL dysfunction has considerable discrimination and calibration abilities.

## Discussion

Radiographs cannot show ACL morphology directly; therefore, a prediction model of ACL function using other anatomical morphologies is significant in clinical practice. Previous studies rarely conducted in-depth or comprehensive analyses of X-ray imaging data [12, 16–18]. We identified position, D2, PTS, and SATT as effective imaging predictors of ACL dysfunction and visualized the combined prediction model using a nomogram. Although the predictive efficiency of position in the validation cohort decreased (Table 3), it did not affect the accuracy of the combined model (Fig. 6). Based on the Youden index, we determined that when the patient’s position was located at the middle and posterior tibial plateau, D2 exceeded 1.40 mm, PTS was greater than 7.90°, and SATT exceeded 4.49 mm. Accurate predictions of ACL dysfunction could be achieved with optimal sensitivity and specificity (Table 3). Our findings significantly alleviate the difficulties that clinicians face in identifying ACL dysfunction using radiographic imaging. Our prediction model enables physicians to quickly, easily, and accurately assess the function of the ACL in KOA patients, rather than injury, which has a positive significance for guiding their subsequent treatment and assessment in the indications of knee replacement surgery.

To screen the imaging variables, we adopted previous studies [12, 16–18, 26–28] and included our original variables, such as osteophyte distribution and wear depth measurements (Tables S1 and S2). ACL abnormalities lead to knee instability and compensatory development of osteophytes around the knee to maintain stability [29]. Therefore, we explored the presence of osteophytes around the knee, which might indicate ACL dysfunction, and found no significant differences in TO and FO between the groups (Table S2). The IO showed a significant difference between the two groups, but had limited predictive value for ACL dysfunction (Table 2). We suggest that IO may act as an important factor in accelerating ACL degeneration caused by physical wear. However, the IO may not be a compensatory manifestation of knee instability because of ACL dysfunction. In contrast, ACL injuries often result in anterior subluxation of the tibia, which increases the risk of damage to the bone and cartilage in the posterolateral compartment [30, 31]. Numerous studies have identified a steep PTS and excessive anterior tibial translation as predictive risk factors for primary ACL reconstruction [26, 32, 33]. This indicates that the bony relationships and morphology between the tibia and femur likely affect ACL function. The inclusion of the K-L, HKA, CTFS, TSS, PTS, and SATT allowed for a multifaceted assessment of knee morphology and positional relationships (Tables S2 and S3). Although Matsuura et al. [34] found that the degree of deformity in KOA is associated with the extent of ACL tears, this study did not identify indicators reflecting the severity of KOA as meaningful for diagnosing ACL function, such as the K-L grade or HKA. This may be attributed to the inclusion of more comprehensive imaging variables in this study, which allowed the identification of signs with greater diagnostic value for ACLD. Moreover, anteromedial osteoarthritis of the knee is a crucial early stage in KOA, indicated by medial compartment degeneration, intact posterior cartilage, normal ACL function, and preserved lateral compartment cartilage [9]. Therefore, lateral radiographs were used to quantify the anteromedial wear of the tibial plateau in terms of position, D, D1, and D2. We believe that the selection of variables was both innovative and sufficient based on previous studies. Therefore, the predictive model we developed is more convincing. The nomogram constructed using the abovementioned four predictors made the conclusions intuitive and highly actionable in clinical practice. However, owing to the study design, we only included patients classified as K-L grades III to IV, who satisfied the indications for knee replacement surgery. Consequently, the applicability of the results of this study to other populations is limited. However, this does not affect the applicability of the research results to the vast majority of clinical scenarios.

Several previous studies predicted ACL function based on radiography and MRI. Although MRI is useful for determining ACL function [35], lateral radiography has shown higher reliability than MRI and physical examination in patients with KOA [12]. MRI is commonly employed to diagnose acute ACL injuries; however, it has limitations in assessing chronic injuries and functional status. MRI may misdiagnose myxoid changes or degeneration caused by ACL when trying to repair itself as ACL dysfunction. Furthermore, loss of cartilage can lead to a relaxed appearance of the functionally intact ACL on MRI, resulting in the misinterpretation of its function [13]. Consequently, the Oxford UKA manual recommends using lateral radiography to assess ACL function rather than relying on MRI. Tibial wear locations observed using lateral radiography have been shown to predict ACL function with high accuracy. The persuasiveness of the results is limited by the small sample size, insufficient variables included, lack of validation, or indirect methods of assessing ACL function such as MRI and physical examinations [16, 36, 37]. This study included 272 patients with 13 radiographic features to improve the accuracy of the prediction model. Direct intraoperative observation combined with a tension test (Supplementary Fig. 1) provided more persuasive evidence for ACL diagnosis. Therefore, the results showed good diagnostic efficiency and agreement with internal validation.

The primary limitation of this study is the absence of external validation, which may restrict the generalizability of the results. However, the internal validation outcomes indicated that the findings of this study were reliable for the existing datasets. Future multicenter studies and external validation efforts may corroborate the findings. Second, this study used manual measurements of imaging features by two independent observers. Although clinically practical, it introduced observer variability. Therefore, we will apply artificial intelligence and deep learning-based automated image processing techniques to assist in diagnosing in the future, thereby reducing inter-observer variability.

## Conclusions

The position, D2, PTS, and SATT measured using radiography were identified as effective predictors of ACL dysfunction. The combined prediction model visualized using the nomogram can help physicians easily and accurately predict ACL dysfunction in KOA patients.

## Supplementary Information

Supplementary Material 1: Fig. S1. ACL dysfunction was obtained in the operation. The plan of undergoing UKA changed to TKA. Fig. S2. The flowchart of patient selection. Table S1. Comparison of variables associated with tibial plateau wear. Table S2. Comparison of variables associated with osteophytes and K-L grading. Table S3. Comparison of variables associated with tibiofemoral morphology.

## Data Availability

The datasets used and/or analysed during the current study are available from the corresponding author on reasonable request.

## Abbreviations

KOA: Knee osteoarthritis
ACL: Anterior cruciate ligament
ACLF: ACL-functional
ACLD: ACL-dysfunctional
LASSO: Least absolute shrinkage and selection operator
ROC: Receiver operating characteristic
AUC: Area under the curve
UKA: Unicompartmental knee arthroplasty
TKA: Total knee arthroplasty
MRI: Magnetic resonance imaging
K-L: Kellgren-Lawrence
IO: Intercondylar osteophyte
TO: Tibial osteophyte
FO: Femoral osteophyte
CTFS: Coronal tibiofemoral subluxation
TSS: Tibial spine sign
HKA: Hip-knee-ankle angle
PTS: Posterior tibial slope
SATT: Static anterior tibial translation
ICC: Intra-class correlation coefficient
OR: Odds ratio
CI: Confidence interval
BMI: Body mass index
