# Supplementary material for: Radiographic prediction model based on X-rays predicting anterior cruciate ligament function in patients with knee osteoarthritis
Source: Vis Comput Ind Biomed Art. 2025 Jun 6;8:14. doi: 10.1186/s42492-025-00195-w (PMC12143998; doi:10.1186/s42492-025-00195-w)
Supplement: Supplementary file 1 — Supplementary Material 1: Fig. S1. ACL dysfunction was obtained in the operation. The plan of undergoing UKA changed to TKA. Fig. S2. The flowchart of patient selection. Table S1. Comparison of variables associated with tibial plateau wear. Table S2. Comparison of variables associated with osteophytes and K-L grading. Table S3. Comparison of variables associated with tibiofemoral morphology. [file 42492_2025_195_MOESM1_ESM.docx]

**Supplementary**


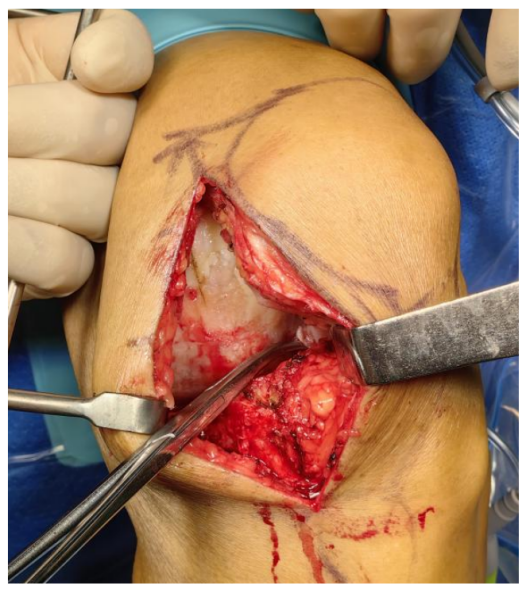


**Fig. S1** ACL dysfunction was obtained in the operation. The plan of undergoing UKA changed to TKA


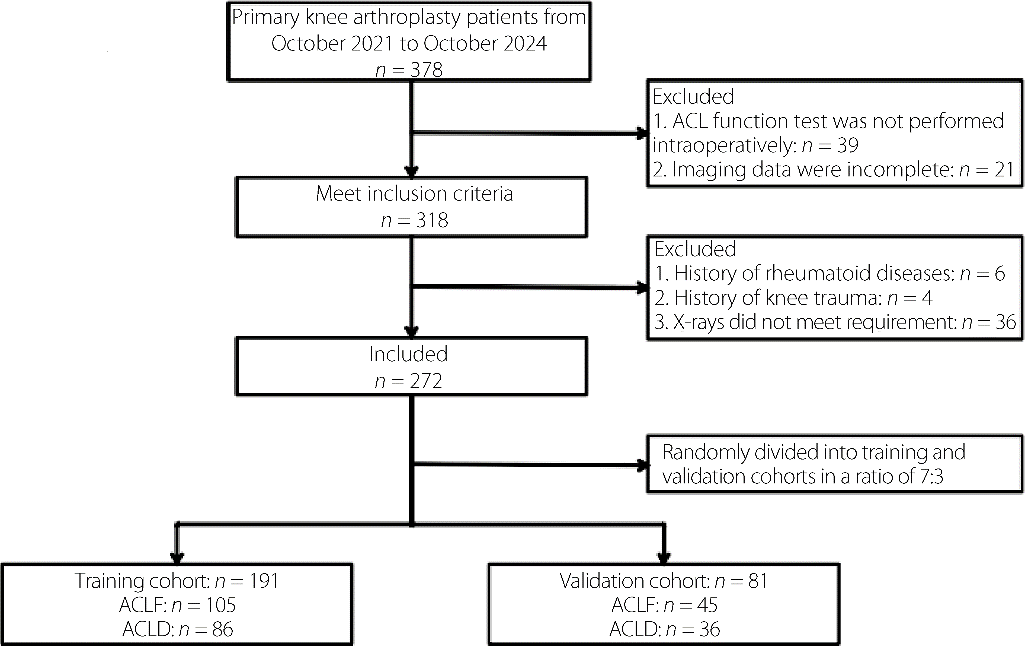


**Fig. S2** The flowchart of patient selection

**Table S1** Comparison of variables associated with tibial plateau wear

| Group | | Position, No. (%) | | | *P* | D (mm) | *P* | D1 (mm) | *P* | D2 (mm) | *P* |
| --- | --- | --- | --- | --- | --- | --- | --- | --- | --- | --- | --- |
|  |  | Anterior | Middle | Posterior |  |  |  |  |  |  |  |
| Training | ACLF | 52 (49.5%) | 42 (40%) | 11 (10.5%) | < 0.01 | 2.02 ± 0.64 | 0.111 | 1.28 ± 0.52 | 0.905 | 1.08 ± 0.52 | < 0.01 |
|  | ACLD | 16 (18.6%) | 51 (59.3%) | 19 (22.1%) |  | 2.21 ± 0.75 |  | 1.36 ± 0.64 |  | 1.65 ± 0.79 |  |
| Validation | ACLF | 24 (53.3%) | 17 (37.8%) | 4 (8.9%) | 0.161 | 2.01 ± 0.59 | < 0.01 | 1.23 ± 0.52 | 0.052 | 1.07 ± 0.51 | < 0.01 |
|  | ACLD | 12 (33.3%) | 18 (50.0%) | 6 (16.7%) |  | 2.38 ± 0.57 |  | 1.51 ± 0.64 |  | 1.42 ± 0.58 |  |

**Table S2** Comparison of variables associated with osteophytes and K-L grading

| Group | | With TO, No. (%) | *P* | With FO, No. (%) | *P* | With IO, No. (%) | *P* | K-L, No. (%) | | *P* |
| --- | --- | --- | --- | --- | --- | --- | --- | --- | --- | --- |
|  |  |  |  |  |  |  |  | Ⅲ | Ⅳ |  |
| Training | ACLF | 51 (48.6%) | 0.241 | 49 (46.7%) | 0.203 | 45 (42.9%) | < 0.01 | 60 (57.1%) | 45 (42.9%) | 0.140 |
|  | ACLD | 50 (58.1%) |  | 49 (57.0%) |  | 57 (66.3%) |  | 39 (45.3%) | 47 (54.7%) |  |
| Validation | ACLF | 24 (53.3%) | 0.822 | 24 (53.3%) | 0.999 | 15 (33.3%) | 0.012 | 24 (53.3%) | 21 (46.7%) | 0.412 |
|  | ACLD | 21 (58.3%) |  | 19 (52.8%) |  | 23 (63.9%) |  | 15 (41.6%) | 21 (58.3%) |  |

**Table S3** Comparison of variables associated with tibiofemoral morphology

| Group | | HKA (°) | *P* | CTFS (mm) | *P* | PTS (°) | *P* | SATT (mm) | *P* | TSS, No. (%) | | | *P* |
| --- | --- | --- | --- | --- | --- | --- | --- | --- | --- | --- | --- | --- | --- |
|  |  |  |  |  |  |  |  |  |  | 1 | 2 | 3 |  |
| Training | ACLF | 8.30 ± 1.89 | < 0.01 | 3.98 ± 1.02 | 0.516 | 7.43 ± 0.92 | 0.015 | 4.57 ± 1.05 | < 0.01 | 59 (56.2%) | 29 (27.6%) | 17 (16.2%) | 0.359 |
|  | ACLD | 9.24 ± 2.05 |  | 3.88 ± 1.14 |  | 8.02 ± 1.52 |  | 5.30 ± 1.13 |  | 41 (47.7%) | 32 (37.2%) | 13 (15.1%) |  |
| Validation | ACLF | 8.60 ± 1.91 | 0.299 | 3.72 ± 1.28 | 0.549 | 7.41 ± 1.04 | < 0.01 | 4.23 ± 0.92 | <0.01 | 26 (57.8%) | 14 (31.1%) | 5 (11.1%) | 0.289 |
|  | ACLD | 9.00 ± 1.57 |  | 3.57 ± 1.30 |  | 8.69 ± 1.59 |  | 5.39 ± 1.32 |  | 24 (66.7%) | 6 (16.7%) | 6 (16.7%) |  |
